# Current News

**Published:** 1892-10

**Authors:** 


### Current News.

### THE WORLD'S CONGRESS AUXILIARY OF THE WORLD'S COLUMBIAN EXPOSITION.

CHARLES C. BONNEY, *President.* LYMAN J. GAGE, *Treasurer.*
THOMAS B. BRYAN, *Vice-President.* BENJ. BUTTERWORTH, *Secretary.*

### DEPARTMENT OF MEDICINE.

#### GENERAL DIVISION OF DENTAL AND ORAL SURGERY.

*Preliminary Address of the Committee on a Dental Congress.*

It is the aim of the World's Columbian Exposition to gather together the evidences of the material progress and achievement of the civilization of the world, and so arrange them that every department of human endeavor may be studied and examined through all its various grades of development.

It is also their desire to represent the intellectual and scientific development and achievement of the entire civilized world by a series of great Congresses, to be held during the progress of the Exposition.

In pursuance of this object the World's Congress Auxiliary was organized by the World's Columbian Exposition, and it has received the recognition and support of the government of the United States.

It is the plan of the World's Congress Auxiliary to bring into communication, through these Congresses, the best thinkers and workers in every department of knowledge, including Religion, Science, Philosophy, Literature, Art, Agriculture, Trade, Labor, etc., and by the presentation and interchange of ideas, methods, theories, and practical experiences to promote the advancement of all that is noblest and best of our present civilization.

Committees have therefore been appointed to organize a series of Congresses, representing nearly every field of thought, and of speculative and practical endeavor.

In the field of professional achievement, Medicine and Surgery, in their various special applications, will form a very large and interesting feature of the work of the World's Congress Auxiliary.

Dentistry is an important department of Medical Science, and an outgrowth of our modern civilization. Its present perfection is in considerable degree due to the thought and labor of American minds.

The history of modern dentistry is covered by a period of less than two generations, and yet it has advanced from the rude operations practised by the blacksmiths and barbers to one of the most scientific and exact of the specialties of the healing art.

Scientific Dentistry had its birth in the United States of America. This country has the proud distinction of having organized the first school for the teaching of dental science, and the establishment of the first periodical journal devoted to the interests of dentistry, while very many of the most useful appliances and scientific methods have originated on this side of the Atlantic.

It is therefore eminently fitting that Dentistry be represented at the World's Columbian Exposition by a display of the progress which has been made in the development of its materials, instruments, appliances, processes, and methods of a practical nature, and in scientific research, literature, and professional education.

With this end in view the dentists of the United States took steps in August, 1890, to organize such a World's Congress, by the appointment of a General Executive Committee, to whom the whole matter of organizing and conducting the Congress was referred.

The work therefore of the Committee on Dental Congresses, appointed by the World's Congress Auxiliary, will be chiefly in co-operation with that General Executive Committee, in publishing to the world from time to time the progress of the work of organization, in promoting the interests of the Congress in every way within their power, and keeping it in harmony with the general plans of the World's Congress Auxiliary.

Every effort will be made to secure the best talent in the presentation of scientific subjects, and in practical demonstrations.

The World's Columbian Exposition, through its Directory, will provide ample accommodations for all the various World's Congresses to be held in Chicago in 1893. The Memorial Art Palace, now in process of erection upon the shore of Lake Michigan, and located near the centre of the city, will be devoted to this purpose. This building will contain two large audience-rooms, with a seating capacity of about three thousand each, which will be used for the general Congresses of the various departments, besides numerous smaller rooms, suitable for the Chapters and Sections of the Congresses, thus affording for the Dental Congress ample accommodations for clinical demonstrations of a suitable nature.

During the sessions of the Dental Congress several popular evening meetings will be held, to which the general public will be invited. At these meetings, which are intended to be educational, illustrated lectures will be delivered by some of the most eminent men of the profession upon topics which are deemed to be of vital importance to the public. These meetings will be especially under the control and management of the World's Congress Auxiliary. When the suggestions of the Advisory Councillors of the Dental Congress shall have been received as to the most interesting and vital questions to be presented, a programme will be arranged for publication.

A cordial invitation is extended to the dentists of the world to take part in the scientific work of the Congress by the presentation of papers and discussions or demonstrations of new or improved methods and appliances.

America, and Chicago in particular, will have a hearty welcome for all who may come.

An earnest effort was made to bring the meeting of this Congress in close connection with others of the Department of Medicine, but that effort having proved unavailing, arrangements have been effected under which the meeting of the dental profession will be held on or near August 17, and is expected to continue during the week or ten days following. Definite dates and details will be given in the programme.

Communications in reference to the special work of the Congress should be addressed to DR. A. O. HUNT, Secretary World's Columbian Dental Congress, Iowa City, Iowa, U.S.A.

Communications in reference to the general work of the World's Congress Auxiliary and suggestions from the Advisory Councillors may be addressed to the Chairman of the Committee.

DR. JOHN S. MARSHALL, *Chairman*,
No. 34 Washington Street, Chicago.

DR. A. W. HARLAN, *Vice-Chairman.*

Dr. G. V. Black, Dr. C. N. Johnson, Dr. George H. Cushing,
Dr. N. Nelson, Dr. A. E. Baldwin, Dr. A. W. Freeman,
Dr. E. S. Talbot, Dr. George A. Christman,

*Committee of the World's Congress Auxiliary on a Dental Congress.*

THE WOMAN'S COMMITTEE OF THE WORLD'S CONGRESS AUXILIARY ON A DENTAL CONGRESS.

DR. HATTIE E. LAWRENCE, *Chairman.*
DR. MARIE T. BACON, *Vice-Chairman.*
Dr. Emma Beanham, Dr. Louise Peterson, Dr. Rebecca McIntosh.
WORLD'S CONGRESS HEAD-QUARTERS, CHICAGO, June, 1892.

PARTIAL LIST OF THE ADVISORY COUNCIL OF THE WORLD'S CONGRESS AUXILIARY ON A DENTAL CONGRESS.

Dr. W. D. Miller, Berlin, Germany; Dr. F. Busch, Berlin, Germany; Dr. Thos. W. Evans, Paris, France; Dr. E. Magitot, Paris, France; Dr. G. W. Sparrock, Lima, Peru; Dr. W. B. Macleod, Edinburgh; Dr. A. W. W. Baker, Dublin; Dr. Ernst Sjoberg, Stockholm, Sweden; Dr. Charles S. Tomes, London, England; Dr. W. H. Coffin, London, England; Dr. W. Geo. Beers, Montreal, Canada; Dr. H. C. Edwards, Madrid, Spain; Dr. E. Lecaudy, Paris, France; Dr. —— Plattschick, Pavia, Italy; Dr. Joseph Arkövy, Buda-Pesth, Hungary; Dr. C. Redard, Geneva, Switzerland; Dr. J. G. Van Marter, Rome, Italy; Dr. W. H. Morgan, Nashville, Tenn.; Dr. W. H. Dwinelle, New York City; Dr. R. B. Winder, Baltimore, Md.; Dr. Elisha G. Tucker, Boston, Mass.; Dr. W. W. H. Thackston, Farmville, Pa.; Dr. J. B. Rich, Washington, D. C.; Dr. J. D. White, Philadelphia, Pa.; Dr. W. H. Eames, St. Louis, Mo.; Dr. J. B. Patrick, Charleston, S. C.; Dr. C. C. Knowles, San Francisco, Cal.; Dr. F. J. S. Gorgas, Baltimore, Md.; Dr. G. V. Black, Jacksonville, Ill.; Dr. J. E. Garretson, Philadelphia, Pa.; Dr. R. Finlay Hunt, Washington, D. C.; Dr. E. Bacon, Portland, Me.; Dr. Benjamin

Lord, New York City ; Dr. A. L. Northrop, New York City ; Dr. W. W. Allport, Chicago, Ill. ; Dr. W. W. Walker, New York City ; Dr. L. D. Carpenter, Atlanta, Ga. ; Dr. J. Y. Crawford, Nashville, Tenn. ; Dr. W. J. Barton, Paris, Texas ; Dr. J. Taft, Cincinnati, Ohio ; Dr. C. S. Stockton, Newark, N. J. ; Dr. L. D. Shepard, Boston, Mass. ; Dr. H. J. McKellops, St. Louis, Mo. ; Dr. A. O. Hunt, Iowa City, Iowa ; Dr. H. B. Noble, Washington, D. C. ; Dr. Geo. W. McElhaney, Columbus, Ga. ; Dr. J. C. Storey, Dallas, Texas ; Dr. M. W. Foster, Baltimore, Md. ; Dr. A. W. Harlan, Chicago, Ill. ; Dr. J. S. Marshall, Chicago, Ill.

PARTIAL LIST OF THE WOMAN'S ADVISORY COUNCIL ON A DENTAL CONGRESS.

Dr. Lucy Hobbs Taylor, Lawrence, Kansas ; Dr. Olga Neymann, New York City ; Dr. Jessie Ritchie, Des Moines, Iowa ; Dr. Annie D. Ramborger, Philadelphia, Pa. ; Dr. Jennie Hilton, Fort Atkinson, Wis. ; Dr. Clara W. McNaughton, Washington, D. C. ; Dr. Kate C. Moody, Mendota, Ill. ; Dr. Martha J. Robinson, Cleveland, Ohio ; Dr. Annie F. Reynolds, Boston, Mass. ; Dr. Mary T. Benfield, Honolulu, Hawaii ; Dr. Marie Holst, Aarhuus, Denmark ; Dr. Henriette Tiburtius-Hirschfeld, Berlin, Germany ; Dr. Helene Wongl v. Swiderska, St. Petersburg, Russia ; Dr. Bella Meller, Vienna, Austria ; Dr. Helene Freudenheim, Königsberg, Germany ; Dr. Marie M. Schneeyans, Elberfeld, Germany ; Dr. Emma Lacey, London, England ; Dr. Clotilde Lenta, Rome, Italy.

---

### MISSOURI STATE DENTAL ASSOCIATION.

THE Twenty-seventh Annual Meeting of the Missouri State Dental Association was held at Clinton, Mo., July 5 to 8, inclusive. There were twenty-six new members admitted, and eleven papers read.

The following is the list of officers and committees elected for the ensuing year:

President, J. D. Patterson, Kansas City ; First Vice-President, W. E. Tucker, Springfield ; Second Vice-President, DeCoursey Lindsley, St. Louis ; Corresponding Secretary, William Conrad, St. Louis ; Recording Secretary, H. A. Rubey, Clinton ; Treasurer Jas. A. Price, Weston.

*Executive Committee.*—C. B. Hewitt, Kansas City; A. J. McDonald, Kansas City; J. E. Crozier, Lees Summit.

*Board of Censors.*—E. E. Shattuck, Kansas City; H. A. Cress, Warrensburg; E. B. Crane, California.

*Committee on Ethics.*—Frank Slater, Rich Hill; J. B. Newby, St. Louis; C. L. Hungerford, Kansas City.

*Committee on Law.*—Jas. A. Price, Weston.

*Committee on Publication.*—J. E. Crozier, Lees Summit; T. J. Frey, Moberly; C. L. Hickman, St. Louis.

*Committee on New Appliances.*—C. L. Hungerford, Kansas City.

Supervisor of Clinics, C. H. Darby, St. Joseph.

The next meeting of the Association will be held at Excelsior Springs, Mo., on the first Tuesday after July 4, 1893.

William Conrad,
*Corresponding Secretary.*

St. Louis, Mo.

---

### RECENT PATENTS.

A list of recent patents, reported specially for the International Dental Journal.

479,407.—Dental Syringe. John M. Drennan, New York, N. Y. assignor to the S. S. White Dental Manufacturing Company, Philadelphia, Pa. Filed June 2, 1892.

480,121.—Dental Disk-Carrier. Mathew S. Nichols, Central Village, Conn. Filed June 25, 1891.

480,261.—Dental Cabinet. Christian A. Herr, Osborn, Ohio. Filed August 24, 1891.

480,357.—Dental Jaw-Brace. Arthur E. Worthen, Melrose, Mass., assignor of one-half to Charles L. Sprague, same place. Filed April 12, 1892.

480,471.—Dental Moistening-Pad. George W. Melotte, Ithaca, N. Y. Filed April 30, 1892.

480,678.—Dental Engine. Charles W. Thomas, Jersey City, N. J., assignor of five-sixths to Philip Van Volkenburgh, New York, and John H. Pendleton, Brooklyn, N. Y. Filed October 21, 1890.

Designs. 21,771. Dental Punch. Charles E. Blake, Sr., San Francisco, Cal. Filed June 27, 1892. Term of patent fourteen years.

### CONNECTICUT VALLEY DENTAL SOCIETY.

At the Twenty-eighth Annual Meeting of the Connecticut Valley Dental Society, held at Greenfield, Mass., June 1, 2, and 3, the following officers were elected for the ensuing year:

President, Dr. F. W. Williams, Greenfield, Mass.; Vice-Presidents, Dr. W. H. Rider, Danbury, Conn., Dr. W. O. Barrett, Ware, Mass.; Secretary, Dr. Geo. A. Maxfield, Holyoke, Mass.; Assistant Secretary, Dr. A. J. Cutting, Southington, Conn.; Treasurer, Dr. F. R. Rice, North Adams, Mass.

Geo. A. Maxfield, D.D.S.,
*Secretary.*

---

### AMERICAN ACADEMY OF DENTAL SCIENCE.

The Twenty-fifth Annual Meeting of the American Academy of Dental Science will be held in Boston on Wednesday, November 16, 1892. The Annual Address will be delivered by Dr. A. W. Harlan, of Chicago. As this will be the Twenty-fifth Anniversary of the Academy, a full attendance of members (active, associate, and honorary) is particularly requested.

E. N. Harris,
*Corresponding Secretary.*

248 Boylston Street, Boston.

---

### OFFICERS OF THE MASSACHUSETTS DENTAL SOCIETY, 1892–93.

President, J. W. Ball, D.D.S., Boston; Vice-Presidents, W. E. Page, D.M.D., Boston, and Jos. King Knight, D.D.S., Hyde Park; Secretary, Edgar O. Kinsman, D.D.S., Cambridge; Treasurer, Edward Page, M.D., D.M.D., Charlestown; Librarian, Jos. King Knight, D.D.S., Hyde Park.

*Executive Committee.*—Geo. F. Eames, M.D., D.D.S., Boston; W. E. Boardman, D.M.D., Boston; H. S. Draper, D.D.S., Boston; J. H. Daly, D.D.S., Boston; T. W. Clements, D.D.S., Brookline.

Edgar O. Kinsman,
*Secretary.*

### NEW ENGLAND DENTAL SOCIETY.

CAMBRIDGE, MASS., September 17, 1892.

THE Thirtieth Annual Meeting will be held in the lecture-room of the Natural History Society Building, corner of Berkeley and Boylston Streets, Boston, Mass., Thursday, October 6, and at the Boston Dental College, Friday, October 7. Programmes will be sent later. A good meeting is promised by the Executive Committee. Mark off the time on your appoinment-books *now*, and endeavor to attend.

EDGAR O. KINSMAN, D.D.S.,
*Secretary.*

